# Perception and beliefs about mental illness among adults in Karfi village, northern Nigeria

**DOI:** 10.1186/1472-698X-4-3

**Published:** 2004-08-20

**Authors:** Mohammed Kabir, Zubair Iliyasu, Isa S Abubakar, Muktar H Aliyu

**Affiliations:** 1 Department of Community Medicine, Aminu Kano Teaching Hospital, Kano, Nigeria & Department of Community Medicine & Primary Care, Bayero University, Kano, Nigeria; 2 Department of Epidemiology, School of Public Health, University of Alabama at Birmingham, USA

**Keywords:** Mental illness, perception, beliefs, attitudes, northern Nigeria

## Abstract

**Background:**

This study was designed to examine the knowledge, attitude and beliefs about causes, manifestations and treatment of mental illness among adults in a rural community in northern Nigeria.

**Methods:**

A cross sectional study design was used. A pre-tested, semi-structured questionnaire was administered to 250 adults residing in Karfi village, northern Nigeria.

**Results:**

The most common symptoms proffered by respondents as manifestations of mental illness included aggression/destructiveness (22.0%), loquaciousness (21.2%), eccentric behavior (16.1%) and wandering (13.3%). Drug misuse including alcohol, cannabis, and other street drugs was identified in 34.3% of the responses as a major cause of mental illness, followed by divine wrath/ God's will (19%), and magic/spirit possession (18.0%). About 46% of respondents preferred orthodox medical care for the mentally sick while 34% were more inclined to spiritual healing. Almost half of the respondents harbored negative feelings towards the mentally ill. Literate respondents were seven times more likely to exhibit positive feelings towards the mentally ill as compared to non-literate subjects (OR = 7.6, 95% confidence interval = 3.8–15.1).

**Conclusions:**

Our study demonstrates the need for community educational programs in Nigeria aimed at demystifying mental illness. A better understanding of mental disorders among the public would allay fear and mistrust about mentally ill persons in the community as well as lessen stigmatization towards such persons.

## Background

Throughout the world, there is an increasing awareness of mental illness as a significant cause of morbidity [1]. This awareness has increased with the steady decline of morbidity due to nutritional disorders, communicable diseases and other forms of physical illness, especially in countries undergoing epidemiological transition [2].

Mental and behavioral disorders are common, affecting more than 25% of all people at some time during their lives [3]. They are also universal, affecting people of all countries and societies, regardless of age, gender and income. The point prevalence of mental illness in the adult population at any given time is about 10% [3]. Similarly, around 20% of all patients seen by primary health care providers have one or more mental health disorders [3].

The role of the community in the prevention and care of the mentally handicapped has now been widely acknowledged and is regarded as the most appropriate basis for the development of mental health programs. Several studies have shown that knowledge of public attitude to mental illness and its treatment is a vitally important prerequisite to the realization of successful community-based programs [4-6]. The recognition of mental disorder also depends on a careful evaluation of the norms, beliefs and customs within the individual's cultural environment. Furthermore, community attitude and beliefs play a role in determining help-seeking behavior and successful treatment of the mentally ill. Unarguably, ignorance and stigma prevent the mentally ill from seeking appropriate help.

People tend to have strong beliefs about the mentally ill, and many of these concepts are based on prevailing local systems of belief [7]. In developing any mental health education program, the basis of such beliefs must be taken into consideration. People's beliefs regarding mental illness should not only be known, but the purpose of their beliefs should be understood. Such attitudes and beliefs about mental illness can only be studied within a cultural context. Although the knowledge and perception of mentally ill patients and their relatives regarding mental illness has been reported from southwest Nigeria [8], to date there is little research on public attitudes towards mental illness from northern Nigeria, a culturally distinct part of the country. This paper is therefore one of the first to report findings related to attitudinal research on mental illness from northern Nigeria.

The 1995 Nigeria National Mental Health Policy advocates the integration of mental health promotion, treatment and rehabilitation into primary health care services (PHC). However, this goal cannot be successfully achieved without an understanding of community attitudes towards mental illness. We therefore set out to ascertain the perceptions, attitudes and beliefs of adults regarding the causes, manifestations and treatment options of mental illness in a traditional Hausa community near Kano, Nigeria.

## Methods

### Study area

The study population included 250 adults residing at Karfi village, about 15 kilometers from Kano city. This typical Hausa community has 11,314 inhabitants [9]. Majority are Muslims preoccupied with farming and petty trading. About 32.0% of the populace are literate. The village has one primary health centre and several traditional healers. Referrals from the health centers are sent to Kura General Hospital and occasionally to Aminu Kano Teaching Hospital. Informed consent was obtained from respondents prior to commencement of the interviews.

### Study design

A cross sectional descriptive study

### Sample size and sampling technique

A multistage sampling technique was adopted. In the first stage, four wards out of the seven in the village were randomly selected. After house numbering, 250 houses were selected from the four wards using a table of random numbers. Where more than one household was found in a house, one household was selected by balloting. Finally, one adult was selected at random from each household for the interview. A final sample size of 250 adults was therefore obtained.

### Instrument description/ Data collection

We adopted and modified a pre-existing semi-structured questionnaire [7] to evaluate the perceptions and beliefs of adults in Karfi village. The questionnaire was in three parts; the first section inquired about personal data including age, sex, ethnicity, religion, marital status, educational level and occupation; the second part elicited awareness of existence of mental illness in the community, knowledge of causal factors, manifestation of the disorder and awareness and preference of treatment options, while the third part explored the attitudes, beliefs and perception of the respondent towards the mentally ill. Attitudes such as fear, avoidance, anger, suspicion, mistrust, hostility were considered negative, whereas sympathetic attitude, willingness to care for a mentally ill relative or friend and tolerance were considered positive attitudes.

The study instrument was validated using a pilot study of 10 randomly selected households in a nearby village with similar demographic characteristics (Kumbotso). Results of the pilot study were used to modify content and wording of the questionnaire. Previously trained medical undergraduates fluent in Hausa language administered the questionnaires to the sample population.

### Data analysis

The data was analyzed using the Epi-Info^® ^6.0 statistical software package (CDC Atlanta, Georgia, USA). Descriptive statistics were depicted using absolute numbers, simple percentages, range, and measures of central tendency (mean, median) as appropriate. The Chi-square test was used to test the significance of associations between categorical groups. All tests of hypothesis were two-tailed with a type 1 error rate fixed at 5%.

## Results

In all, 250 respondents were interviewed with 167 males and 83 females giving a sex ratio of 2:1 in favor of males. Their ages ranged from 18 to 74 years. Majority (78.0%) of the respondents were aged between 25 and 60 years. The median age (± SD) for the respondents was 34.5 ± 4.6 years; 36.0 ± 2.3 years for males and 28.5 ± 1.6 years for females. The Hausa-Fulani ethnic group constituted 89% of respondents, and the rest were Yoruba 2.0%, Igbo 5.0% and other minority Nigerian tribes 4.0%. About 16.0% of respondents had primary education, 12.0% had secondary education, and 4.0% had tertiary (post-secondary school) education. Approximately 27.0% of the population sampled had no formal education. Nevertheless, 41.0% of all respondents had Quranic education. The majority of respondents (90.0%) were Muslims and the remaining 10.0% were Christians. About 10.0% were single, 80.0% were married, 6.0% were divorced and the remaining 4.0% were widowed. Forty eight per cent of the respondents were engaged in farming, 25.0% were full-time housewives, 20.0% were engaged in petty trading, 7.0% were students and the remaining 4.0% were civil servants. About 13 persons interviewed (5.2%) had a relative with mental illness, but because of the small sample size we did not separately analyze findings from this group of participants.

The most common symptoms proffered by respondents as manifestations of mental illness (Table 1) included aggression/destructiveness (22.0%), talkativeness (21.2%), eccentric behaviour (16.1%), and wandering (13.3%). Drug misuse in form of alcohol ingestion, cannabis and other psychoactive street drugs were identified as major causes of mental illness (34.3%), followed by effect of divine wrath or God's will (18.8%), magic or spirit possession (18.0%), and accidents/trauma (11.7%) (Table 2). Heredity, family conflicts and financial distress/poverty were uncommon responses.

**Table 1:** Respondents' perceived manifestations of mental disorder

**Manifestation**	No.* (%)	**Rank order**
Aggression/destructiveness	173 (22.0)	1
Talkativeness	167 (21.2)	2
Eccentric behavior	127 (16.1)	3
Wandering	105 (13.3)	4
Self-neglect	86 (10.9)	5
Nudity	56 (7.1)	6
Restlessness/anxiety	50 (6.4)	7
Insomnia	15 (1.9)	8
Loss of consciousness	8 (1.0)	9

* Multiple responses recorded. Percentages represent proportions of responses obtained.

**Table 2:** Perceived causes of mental illness

**Perceived cause**	No.* (%)	**Rank**
Misuse of drugs^†^	88 (34.3)	1
Divine punishment, God's will	48 (18.8)	2
Magic, spirit possession	46 (18.0)	3
Accidents/trauma	30 (11.7)	4
Heredity	27 (10.5)	5
Family conflicts/marital disharmony	14 (5.5)	6
Financial distress/poverty	3 (1.2)	7

*Multiple responses recorded. ^† ^Include street drugs and alcohol.

The majority of respondents (46.0%) opted for orthodox medical care when asked about preferred source of treatment for the mentally ill. This was followed by spiritual healing (exorcism) (34.0%) and the use of traditional herbal medicines (18.0%) (Table 3).

**Table 3:** Respondents' preferred treatment for mental illness

**Response**	**No. (%)**
Orthodox Medicine	116 (46.0)
Traditional Medicine	46 (18.0)
Spiritual Healing	85 (34.0)
Others	3 (2.0)
Total	250 (100.0)

Table 4 shows that majority of the respondents harbored negative feelings towards the mentally ill, mainly in the form of fear (n = 113) and avoidance (n = 81). A total of 117 respondents (46.8%) were sympathetic towards the plight of the mentally sick with female respondents showing more inclination for sympathy compared to their male counterparts. The female respondents, however, tend to be fearful and avoid the mentally sick more than their male counterparts.

**Table 4:** Distribution of attitude towards the mentally ill by gender

**Attitude**	**Male**	**Female**	**Total**
	No. (%)	No. (%)	
Fear	24 (20.8)	89 (79.2)	113
Avoidance	12 (14.4)	69 (85.6)	81
Anger	48 (96.8)	2 (3.2)	50
Suspicion	36 (90.4)	4 (9.6)	40
Hostility	32 (93.6)	3 (6.4)	35
Mistrust	28 (89.6)	4 (10.4)	32
Indifference	16 (96.0)	1 (4.0)	17
Sympathy	39 (33.6)	78 (66.4)	117
Kindness	17 (20.8)	62 (79.2)	79

Literacy status was significantly associated with the type of feeling exhibited by the participants. Literate respondents were seven times more likely to exhibit positive feelings towards the mentally ill as compared to non-literate subjects (OR = 7.6, 95% confidence interval = 3.8–15.1) (Table 5).

**Table 5:** Influence of literacy level of respondents on attitude towards the mentally ill

	**Positive attitude**	**Negative attitude**	**Total**	Odds ratio (95%CI)*
Literate	92	15	107	7.6 (3.8–15.1)
Not literate	64	79	143	
Total	156	94	250	

*CI = confidence interval, p < 0.0001

## Discussion

Aggression/destructiveness, talkativeness, and eccentric behaviors were the most frequently mentioned perceived symptoms of mental illness by respondents. This finding suggests that one has to display behaviour that attracts public attention and is therefore socially disruptive, to be recognized as having a mental disorder. This finding is similar to that documented by White in Tanzania [10] and Asuni *et al*. [7] among Yoruba patients in Western Nigeria. It is notable that hallucinations and delusions that are frequently mentioned in the literature as prototypes of gross psychotic states were not mentioned by the respondents as features of mental illness, probably because such features are not as tangible as aggressive attitudes.

Misuse of drugs ranked highest among the respondents as a perceived cause of mental disorders than most of the other traditional etiologies. This finding may not be unconnected with increasing use of illicit drugs among the youth in developing countries. Although drug abuse was acknowledged by Iliyasu and Last [11] in their work on mental illness in Kano, northern Nigeria as a leading cause of drug dependent psychosis, Holzinger and colleagues [12] reported that drugs and alcohol was not considered by schizophrenia patients or their relatives to be a common cause of mental illness. Divine punishment ranked second as a perceived causative factor. This response may not be unconnected with the leading response (drug misuse), as many individuals are of the belief that one evokes supernatural wrath by taking intoxicants thus leading to the development of mental illness [3].

Belief in demons as the cause of mental health problems is a well-known phenomenon in many cultures of the world [13] but in our study this factor was ranked 3^rd ^place by respondents (18% of the responses). Our finding is also in contrast to Adebowale and Ogunlesi [8] who found that "supernatural causes" were the most acceptable etiological factor among both mentally ill patients and their relatives in southwest Nigeria.

Only 1.0% of respondents admitted that financial distress or poverty was a possible cause of mental disorder. Such a low score in the face of the present adverse socio-economic conditions prevailing in Nigeria may be explained by the Hausa cultural cum religious belief in providence and patience in the face of adversity. Other factors have also been reported by investigators as being associated with perceived causes of mental illness among patients and their relatives. Srinivasan and Thara [14] found that patient gender and education, duration of illness, the key relative's education, and the nature of relationship were associated with family beliefs about the cause of mental illness.

There was a higher score on the preference for modern medical care in treating psychiatric illness. Similar changes in attitude towards the modern scientific approach regarding mental disorders was documented by Alem et al [15] in their work on mental illness in Ethiopia and by Iliyasu and Last [11].

Fear and avoidance of the mentally sick was frequent among female respondents. Traditionally, men are expected to be outwardly brave and less submissive towards aggression. The high frequency of negative attitudes among our respondents may be due to the fact that the concept of the mentally sick has an unfavourable public image. It has been shown that people may evaluate mental illness negatively, reject and discriminate against mental patients, and base their views on traditional stereotypes [4,10,16].

Literacy was found to be significantly associated with positive attitude towards the mentally sick. Similar findings were reported by Madianos *et al*. [17] in Greece, and by Alem *et al*. [15] in Ethiopia. A study on community attitudes towards the mentally ill in New Zealand also reported that those who had previous contact with the mentally ill held informed and enlightened views [18]. Interestingly, a recent Hong Kong study reported a generally negative attitude towards the mentally ill despite a fairly good knowledge of mental illness among the respondents [19]. Our results support the hypothesis by Wolff *et al*. [19] that negative attitudes towards the mentally ill are fuelled by a lack of knowledge.

## Conclusions

This study demonstrates the need for educational programs aimed at demystifying mental illness. A better understanding of mental disorders among the public would allay fear and mistrust about mentally ill persons in the community as well as lessen stigmatization towards such persons. Our findings may be of utility to health policy makers in the design of community mental health education programs and community mental health services in existing primary health centers in Nigeria.

## Competing interests

None declared.

## Authors' Contributions

KM and IZ initiated the study, and participated in the field work. IZ and AIS did the preliminary analysis and wrote the draft manuscript. KM, AIS and IZ participated in the design of the study and performed the statistical analysis. KM, IZ, and AIS participated in its design and coordination. AMH, KM and IZ wrote the final version of the draft manuscript. All authors read and approved the final manuscript.

## Pre-publication history

The pre-publication history for this paper can be accessed here:


